# Glia Maturation Factor Beta as a Novel Biomarker and Therapeutic Target for Hepatocellular Carcinoma

**DOI:** 10.3389/fonc.2021.744331

**Published:** 2021-11-02

**Authors:** Wan Sun, Changchang Hu, Tianyu Wang, Juan Wang, Jieping Zhang, Furong Gao, Qingjian Ou, Haibin Tian, Caixia Jin, Jingying Xu, Jingfa Zhang, Guo-Tong Xu, Lixia Lu

**Affiliations:** ^1^ Department of Ophthalmology of Shanghai Tenth’s People Hospital and Laboratory of Clinical Visual Science of Tongji Eye Institute, Tongji University, Shanghai, China; ^2^ Department of Biochemistry and Molecular Biology, School of Medicine, Tongji University, Shanghai, China; ^3^ Department of General Surgery of Guizhou Provincial People’s Hospital, Guizhou, China; ^4^ Tongji University School of Medicine, Shanghai Tenth People’s Hospital of Tongji University, Shanghai, China; ^5^ Department of Pharmacology, School of Medicine, Tongji University, Shanghai, China; ^6^ Department of Ophthalmology, Shanghai First People’s Hospital, Shanghai Jiao Tong University, Shanghai, China

**Keywords:** hepatocellular carcinoma, GMFB, prognosis, bioinformatics, mitochondria function

## Abstract

Hepatocellular carcinoma (HCC) is one of the most common types of cancer. The novel sensitive biomarkers and therapeutic targets are urgently needed for the early diagnosis of HCC and improvement of clinical outcomes. Glia maturation factor-β (GMFB) is a growth and differentiation factor for both glia and neurons and has been found to be tightly involved in inflammation and neurodegeneration conditions. In our study, the expression level of GMFB was significantly up-regulated in patients with HCC and positively co-expression with tumor node metastases (TNM) stage and histopathological grade of HCC. The high expression level of GMFB was remarkably associated with poor overall survival, which mainly occurred in males rather than females. Multivariate analysis revealed GMFB to be an independent prognostic factor for overall survival in patients with HCC. Results of Gene Ontology (GO) and KEGG pathways analysis showed that down-regulation of pathways related to protein translation and mitochondria function were enriched. Protein-protein interaction analysis revealed the central role of mitochondria protein in HCC. The downregulation of genes involved in glycolysis and gluconeogenesis was observed among the co-expression genes of GMFB. Knockdown of GMFB in Hep3B significantly inhibited proliferation, migration, and invasion of Hep3B cells, and also downregulated the expression levels of some of metal matrix proteinase (MMP), increased mtDNA copy number and loss of mitochondrial transmembrane potential. GMFB influences the malignancy rate of HCC possibly through regulation of the expression of MMPs, mtDNA function and glycolysis. We proposed that GMFB was a promising HCC diagnostic and prognostic biomarker and therapeutic target in HCC.

## Introduction

Hepatocellular carcinoma (HCC) is the main component of primary liver cancer, accounting for 75%-85%, and remains the second leading cause of the death rate of cancers worldwide (1, 2). The major risk factors of HCC showed regional differences. Among the most important risk factors, chronic infection with hepatitis B virus (HBV) or hepatitis C virus (HCV), heavy alcohol intake, obesity and type 2 diabetes were highlighted (3). Although HCC can be prevented by the vaccine against HBV, the incidence and mortality of HCC have been increasing rapidly for the past several years worldwide and represents a considerable public health burden (4–8).

Previous studies have identified biomarkers, such as Alpha-fetoprotein (AFP), apyrimidinic endodeoxyribonuclease 1 (APEX1), glypican 3 (GPC3), Golgi protein-73 (GP73), and Dickkopf-1 (DKK1), for HCC diagnosis (9–13). Among them, AFP is considered the gold standard diagnostic marker for HCC. However, its sensitivity and specificity are low and its expression can be influenced by several non-HCC related factors (14). Nevertheless, the early and specific diagnosis of HCC remains challenging.

Molecularly targeted therapeutics are important methods for treating advanced HCC (15). To date, a large number of biomarkers have been served as therapeutic targets for HCC. For instance, as a multi-targeted tyrosine kinase receptor inhibitor Sorafenib exerts its function by targeting CRAF、BRAF、VEGFRl-3、PDGFR-p、cKIT、FLT-3 and RET (16). Although remarkable progress has been achieved, there are no robust biomarkers that could be applied in the early diagnosis and treatment of HCC (17). However, a limited effect with a five-year survival rate of 18% in HCC patients was obtained (18, 19). Therefore, there is an urgent need to develop a novel, highly sensitive and specific biomarker for the diagnosis, prognosis and therapeutic target for HCC (20). There is also a remarkable gender disparity of HCC incidence, primarily males (21). The current study provides evidence that androgen in males could play a critical role in hepatocarcinogenesis and development, and estrogen could protect women from hepatocarcinogenesis (22, 23). Therefore, the gender-related differences should be given special consideration in individual HCC treatment from the perspective of precision medicine.

GMFB is a growth and differentiation factor expressed predominantly in the central nervous system (CNS) and testis (24, 25). As for liver, the expression of GMFB was confirmed by previous proteomics analysis (26, 27). GMFB is involved in growth and differentiation in the vertebrate brain, neutrophil chemotaxis, migration of monocyte, migration, and adherence of T lymphocytes (28–31). GMFB was found to be upregulated in several neuroinflammation and neurodegeneration conditions (32). Increased GMFB expression was found highly associated with multiple types of cancer, including glioma and ovarian cancer, and GMFB overexpression was reported to be co-expression with poor prognosis in ovarian cancer and glioma (33, 34). To date, there have been no studies of the effects of GMFB on HCC, therefore its roles in HCC progression remain unclear.

This study aimed to characterize the association between GMFB and HCC, and reveal a potential underlying mechanism of GMFB of HCC. To verify analysis results, a series of experiments were carried out. The expression level of GMFB affects mtDNA copy number, mitochondrial membrane potential and MMPs expression levels in Hep3B cell, and impairs cell migration, invasion and adhesion. To our knowledge, this is the first study to report the diagnostic and prognostic values of GMFB in HCC and analyze the gender-based correlation between GMFB and overall survival in patients with HCC. Our study provided a new potential diagnostic and prognostic marker, and a novel bio-target for the treatment of HCC.

## Materials and Methods

### Ethics Statement

Our study was approved by the Academic Committee of Tongji University and conducted in accordance with the Declaration of Helsinki. We obtained all the data from the online databases, therefore the informed consent for data collection had already been obtained.

### Oncomine Database Analysis

ONCOMINE (http://www.oncomine.org) is an integrated translational bioinformatics platform composed of datasets and analyses (35). Datasets include samples represented as microarray data measuring either mRNA expression or DNA copy number, which is used to set up analyses on groups of interest like cancer *versus* normal. Oncomine analyses result from computations that are performed on samples within a dataset. To investigate the transcription levels of GMFB in different types of cancers the Oncomine database was used. Student´s t-tests were performed, with results filtered by the cut-off of p-value <0.05, fold change of >1.5 and gene rank in the top 10%.

### UALCAN Database Analysis

UALCAN (http://ualcan.path.uab.edu) is a comprehensive web resource based on 3 RNA-seq databases and the clinical data of 31 cancer types from the The Cancer Genome Atlas (TCGA) database (project ID, TCGA-LIHC) (36). To analyze the levels of the ADF family protein in liver hepatocellular carcinoma. We analyze the transcriptional expression patterns in normal liver tissue and liver hepatocellular carcinoma samples through UALCAN.

### Human Protein Atlas Analysis

HPA (https://www.Proteinatlas.org/) is a valuable tool provided immunostaining on tissues and cell lines as well as differential expression analysis of proteins in normal and tumor tissues (37). In this study, we checked the protein expression of GMFB in the HPA database and analyzed the immunohistochemical results of GMFB in tumor tissues and normal tissues (Antibody: HPA053669).

### cBioPortal

cBioportal (http://www.cbioportal.org/) is an open access powerful tool offered to explore, visualize and analyze multidimensional cancer genomics data. The genomic data include somatic mutations, DNA copy-number alterations (CNAs), mRNA and microRNA (miRNA) expression, DNA methylation, protein abundance and phosphoprotein abundance (38). The Oncoprint module in cBioportal was used to provide a graphic summary of major genetic alterations and changes in gene expression. Increases or decreases in mRNA level were based on a Z-score threshold of 2.0 or more standard deviations from the mean of the reference population. The reference population was the samples that are scored diploid for each gene. In our study, the relationship between GMFB in HCC patients and alteration frequency were analyzed through cBioportal. Venn diagram was created using online Venn software (http://bioinformatics.psb.ugent.be/webtools/Venn/).

### LinkedOmics and GSE

The LinkedOmics database (http://www.linkedomics.org) is an open-access online biometrics platform that contains 32 cancer types comes from 11,158 patients from TCGA database (project ID, TCGA-LIHC) (39). In this study, we determined the GMFB associated gene enrichment using the “LinkInterpreter” module, which performs enrichment analysis based on Gene Ontology, biological pathways, network modules, among other functional categories.

### Metascape, DAVID and Network Analyst Analysis

Metascape and DAVID are online software for gene annotation and gene set enrichment analysis. In this study, Gene Ontology (GO) and Kyoto Encyclopedia of Genes and Genomes (KEGG) pathway enrichment analyses of GMFB co-expression genes were performed using Metascape and DAVID. And the PPI networks were conducted by Metascape. In Metascape, the min overlap was set as 3, the P-value cutoff was 0.01, and the minimum enrichment factor was 1.5. The cutoff value was set as FDR of<0.05. NetworkAnalyst (http://www.networkanalyst.ca) was used to identify the differential expression of GMFB between HCC and normal samples from TCGA database (project ID, TCGA-LIHC) (40).

### Construction of Prognostic Models and Survival Analysis

The Kaplan–Meier Plotter database (http://kmplot.com/analysis/) is an online public database, to draw survival plots using publicly available data (41). In this work, our survival analysis was carried on the Kaplan–Meier Plotter database contains survival information for 364 patients with HCC, 250 male patients and 121 female patients with the “Auto select best cutoff” option. The expression levels of GMFB and clinical features were merged to find independent prognostic factors through univariate and multivariate Cox regression analysis. The receiver operating characteristics curve (ROC) analysis was performed using the ‘survival ROC’ package in R. The area under the ROC curve (AUC) was measured for the prediction of GMFB.

### Cell Lines

Hep3B cells purchased from ATCC were used in this study. All cells were maintained in High Glucose Dulbecco’s Modified Eagle Medium (DMEM) supplemented with 10% FBS (Gibco, Thermo Fisher Scientific, Spain) at 37°C and 5% CO_2_. and cultured.

### Cell Transfection

Hep3B cells were transfected using Lipofectamine 2000 (Invitrogen, USA), according to the manufacturer’s instructions. SiRNA and Lipofectamine 2000 were diluted in FBS free DMEM(Gibco) medium and incubated for 5 min, separately. Then mixed together, and incubated for 10-15 min at room temperature (RT) to form the DNA-Lipofectamine complexes. Finally added to the culture plates.

### Western Blot Analysis

The samples were lysed with cell lysis buffer (RIPA Lysis Buffer; CAT: P0013B; Beyotime Institute of Biotechnology) to extract whole-cell protein. Protein concentration was quantified using a BCA kit (CAT: P0012; Beyotime Institute of Biotechnology Jiangsu, China), and 20–50 μg of each protein were separated by SDS-PAGE using a 12.5% SDS-PAGE gel. After transfer to a polyvinylidene fluoride membrane (Millipore, USA), membranes were blocked with 5% skim milk at room temperature for 1 hr and incubated overnight at 4°C with primary antibody against GMFB (1:2000; Cat no:10690-1-AP; Proteintech) and ACTB (1:5000; Cat no: 20536-1-AP; Proteintech). Secondary antibodies used were rabbit anti-goat Ig-HRP (1:5000; Proteintech). The membranes were then incubated with secondary antibodies for 2 hrs. Proteins were visualized using ECL (Millipore). The density of the bands was determined using ImageJ software (USA).

### Wound Healing Assay

Hep3B cells were seeded in 6-well plates and grown to 90% confluence. The confluent cell monolayers were scratched using a sterile 200µl pipette tip and rinsed with PBS to remove scratched cells. Wound closure was observed for 36 hours. The wound closure rate was calculated as follows: wound closure (%) = (area of initial wound-area of final wound)/area of initial wound ×100.

### Transwell Migration and Invasion Assays

For cell invasion analysis, transwell chambers were coated with Matrigel, and the cell migration (without matrigel) assay was also performed. Cell suspensions in FBS free medium were added to upper transwell chambers (24-well, 8-μm pores; BD Labware, USA), While 600 µL DMEM medium supplemented with 10% FBS was added into lower transwell chambers. Cell counting was performed using the cell counter plugin of ImageJ. The migratory capacity of Hep3B cells under various treatments was evaluated through scratch assay.

### Measurement of mtDNA Copy Number

Total DNA (mtDNA and gDNA) was isolated using TIANamp Genomic DNA Kit from TIANGEN (TIANGEN, Beijing, China) according to the manual. Relative mtDNA copy number was quantified by quantitative PCR (qPCR) (42). The quantitative PCR was performed by using SuperReal PreMix Plus (SYBR Green) (TIANGEN, Beijing, China) on a BIO-RAD CFX96 Touch Real-Time PCR Detection System. The mtDNA (D-loop, MT-TL1 and MT-ND1) was normalized to nuclear DNA (NCOA3). The primers and primer sequences of mtDNA-specific primers used were as previously reported (43). The relative fold changes were calculated by 2^−ΔΔCt^.

### Quantitative Real-Time PCR

Total RNA was isolated using the TRIzol reagent (Takara, Dalian, China) strictly according to the instructions. Then RNA was reverse transcribed to cDNA using PrimeScript RT polymerase (Takara, Dalian, China). SYBR Green Master Mix (Tiangen Biotech, China) on a LightCycler 96 Detection System (Roche) was used for RT-qPCR. Data were analyzed using the 2^-ΔΔCt^ method. Oligos were synthesized by Sangon. Primer sequences were obtained from Origene.

### Mitochondrial Membrane Potential

Hep3B cells were seeded onto coverslip placed in 24-well plates and cultured at 37°C in a 5% CO2 incubator. After 24 h, cells were transfected with scramble control siRNA or GMFB siRNA with Lipo2000 (Invitrogen). Scramble siRNA was used as a control. JC1 staining was performed 48 h after transfection. Cells were washed three times with PBS, and then stained with the 2 μM JC-1 dye (Beyotime, Shanghai, China) in the dark for 20 minutes. Cell visualization was performed by a fluorescence microscope.

### Statistical Analysis

Image analysis was performed using Image J. Student t-test and one-way ANOVA were used in the statistical analysis. Data were expressed as means ± SEM. The p-value less than 0.05 was considered statistically significant.

## Results

### Increased Expression of GMFB in Liver HCC

**Figure 1:**
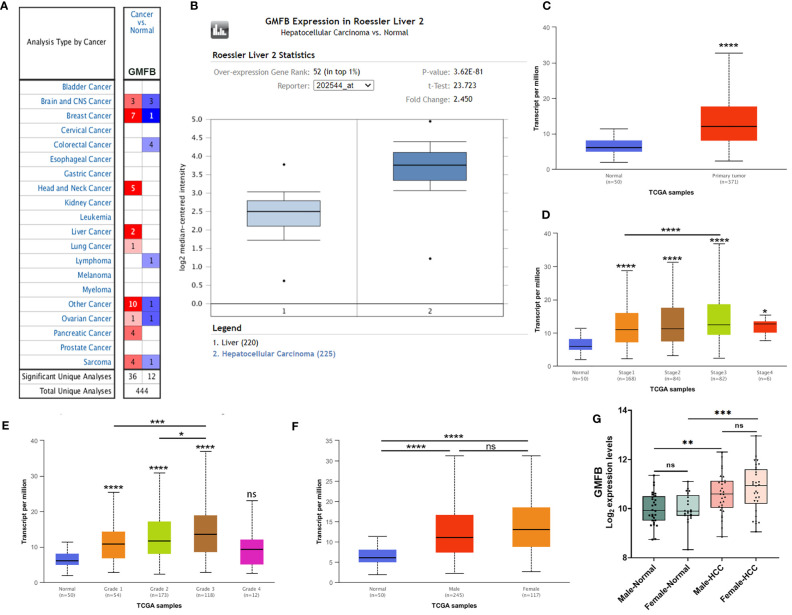
GMFB expression levels in tumors based on sample types, tumor stage, tumor grade. **(A)** The transcription levels of GMFB in different types of cancers from the Oncomine database. The schematic in each cell reveals the numbers of datasets with statistically significant. The cell color is determined by the best gene rank percentile, red indicating high expression and blue indicating low expression. **(B)** The expression of GMFB in HCC and normal liver tissues from the Oncomine database. **(C)** The transcription levels of GMFB in HCC and normal tissues were analyzed by the UALCAN cancer database. The expression of GMFB in HCC is based on tumor stage **(D)**, tumor grade **(E)**, and gender **(F)** from the UALCAN database. **(G)** The transcription levels of GMFB in male normal, female normal, male HCC and female HCC tissues were analyzed by the NetworkAnalyst. *P < 0.05, **P < 0.01, ***P < 0.001, ****P < 0.0001, ns, no significance.

**Figure 2:**
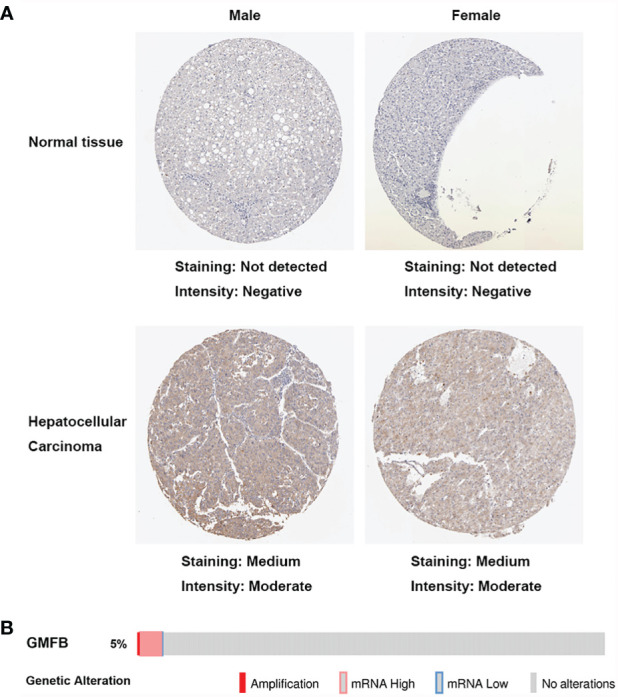
Increased expression of GMFB in liver hepatocellular carcinoma. **(A)** Immunohistochemistry analysis of GMFB protein expression in normal liver tissues and hepatocellular carcinoma tissues of males and females in HPA. **(B)** OncoPrint of GMFB genetic alterations in HCC shows the frequency of genetic alterations included amplification, mRNA upregulation, and mRNA downregulation. The aberrant expression threshold was defined as z−score ±2.0.

We initially used the Oncomine database to analyze the expression profiles, and found that the GMFB transcription levels were markedly higher in breast cancer, head and neck cancer and liver cancer compared to the normal tissues (**Figure 1A**). To study the changes of GMFB mRNA expression levels between HCC and normal liver tissues, we then used the Oncomine and UALCAN database to analyze the differential. The mining of publicly available databases from Roessler Liver 2 Statistics showed that GMFB was up-regulated in HCC (**Figure 1B**). Roessler Liver 2 Statistics was containing 12,624 measured mRNAs from 445 samples. Samples were hybridized to Affymetrix Human Genome HT U133A Array. GEO sample IDs were given in **Supplementary Table 1**. Then, we explored the expression of GMFB in HCC using the UALCAN based on the data resources of The Cancer Genome Atlas database. The results were nearly identical (**Figure 1C**). Furthermore, the analysis of TCGA datasets revealed that the expression of GMFB in HCC was associated with TNM stage 1-3 (**Figure 1D**) and pathological grade 1-3 (**Figure 1E**). Considering the small number of samples of stage 4 and grade 4, the statistical conclusion may not be accurate, and future larger cohort validation is needed. The analysis of TCGA datasets revealed that GMFB was highly expressed in both male and female HCC patients using the UALCAN. And there was no difference was found between male and female HCC patients (**Figure 1F**). To solve the imbalance among normal, male and female data in **Figure 1F**, we downloaded normal (liver), male (HCC) and female (HCC) gene expression data from TCGA further validation. Each sample was assigned a number and 30 samples of each group were randomly selected (**Supplementary Table 2**). The randomization was computer-based, generated in R (version R 3.4.4). The result indicated that there was a significantly higher expression of GMFB in HCC patients in both genders, and no significant gender differences were found in normal (liver) or HCC tissues (**Figure 1G**). Furthermore, Detection of GMFB protein expression in normal and HCC liver tissue of females and males by immunohistochemistry retrieved from the Human Protein Atlas also indicated higher protein expression patterns of GMFB in HCC tissues when compared to normal samples (**Figure 2A**). Furthermore, we also performed GMFB copy number variation (CNV) and mRNA levels among normal and HCC tumor samples using Oncoprint algorithm from cBioPortal, and the result showed that 5% of the HCC cases had undergone genetic changes (**Figure 2B**).

### The Significant Correlation Between Increased GMFB Expression and Poor Overall Survival in HCC

**Figure 3:**
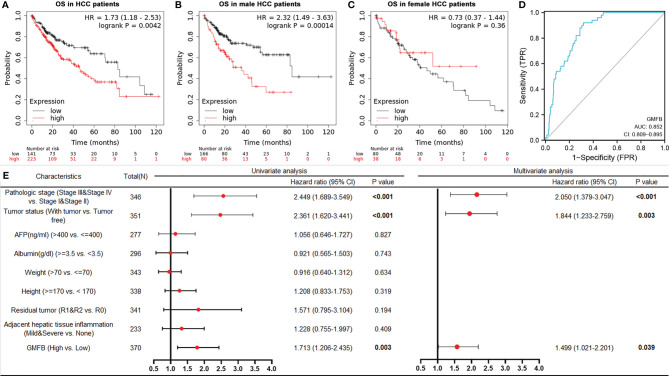
Kaplan-Meier curves showed high mRNA expression levels of GMFB was associated with unfavorable overall survival **(A)** High expression of GMFB was associated with poor survive in HCC patients (KM plot). Kaplan–Meier survival estimation for male **(B)** and female **(C)** HCC patients (KM plot). OS, Overall survival. **(D)** The receiver operating characteristics (ROC) curves for prediction of GMFB expression in patients with hepatocellular carcinomas vs. controls. ROC curves of the radiomics signature, clinical model, and combined model in HCC cohorts **(E)** Univariate and multivariate Cox analysis of the overall survival (OS) by proportional hazard analysis for cancer-specific survival in HCC patients. The red circle indicated the HR, and the transverse lines indicate the 95% CI.

To clarify the associations between the expression levels of GMFB and patients’ clinical outcomes, Kaplan-Meier (KM)Plotter survival analysis was performed. The survival curves indicated that high GMFB expression was significantly associated with poor overall survival (OS) of HCC patients (P=0.0042) (**Figure 3A**). Next, we analyzed the effects of gender differences on the association between GMFB and OS. A significant difference was observed in male patients (**Figure 3B**), but not in females (P=0.36) (**Figure 3C**).

### GMFB Being an Independent Risk Factor for HCC

The AUC values with 95% CI of GMFB was 0.852, CI in a range from 0.809 to 0.895, indicating a significantly diagnostic accuracy for HCC (**Figure 3D**). Univariate analysis by Cox proportional hazard model showed that GMFB, pathologic stage and tumor status were responsible for OS in HCC. Furthermore, the multivariate Cox regression analysis confirmed that GMFB (P=0.039), pathologic stage(P<0.001) and tumor status(P=0.003) were independent factors for predicting the prognosis of HCC patients (**Figure 3E**). The clinical characteristics of the HCC patients were shown in **Supplementary Table 3**.

### The Analysis of Co-Expression Genes With GMFB in HCC Patients

**Figure 4:**
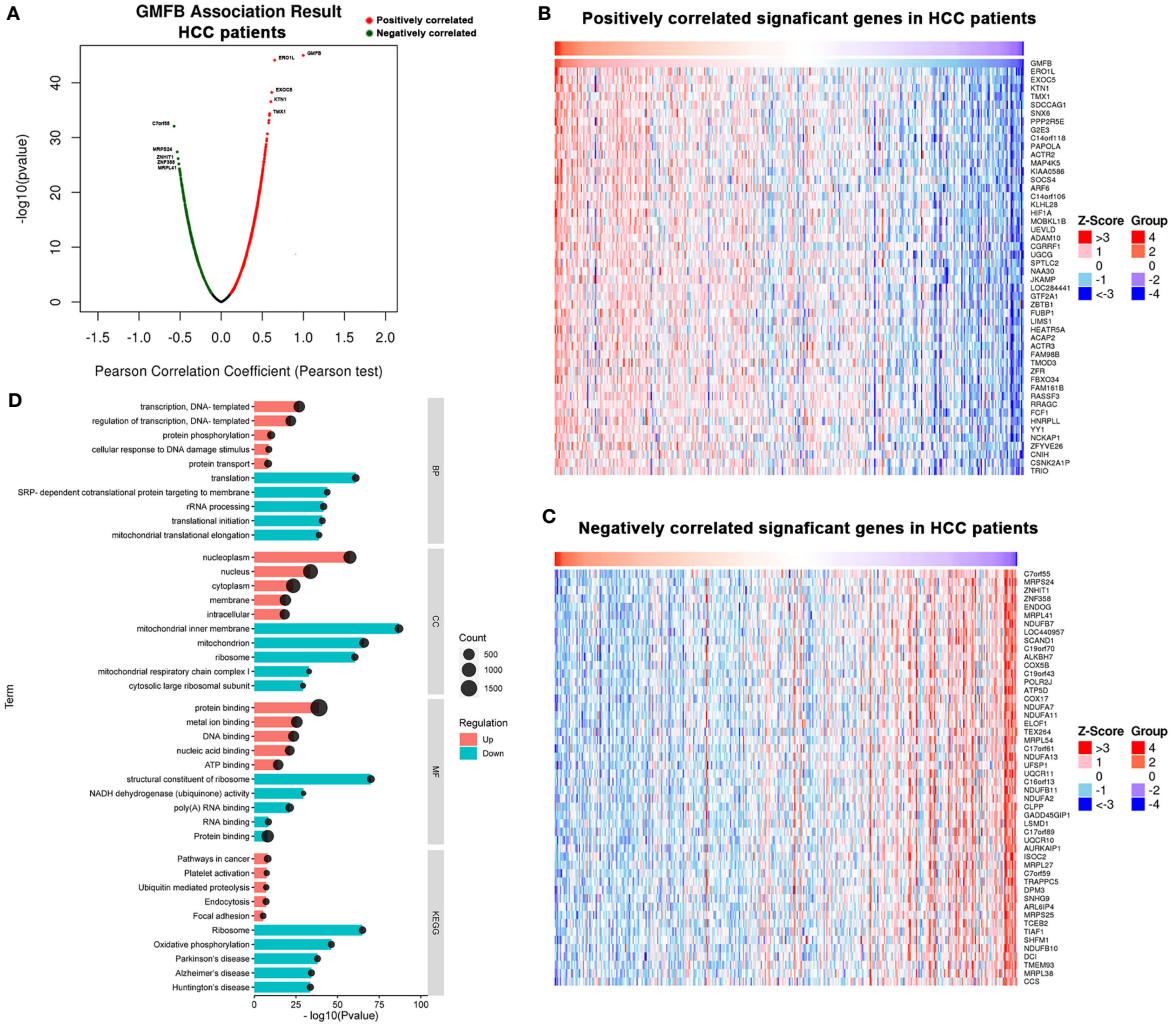
Significant GO terms and KEGG pathways correlated with GMFB in HCC. **(A)** A Pearson test was used to identify the correlations between GMFB differentially expressed in HCC cohort (LinkedOmics). Red dots represent positively significantly correlated genes with GMFB and green dots represent negatively significantly correlated genes with GMFB. Heat maps showing genes positively **(B)** and negatively **(C)** correlated with GMFB in all HCC (TOP 50). **(D)** Enriched GO terms (BP, CC and MF) and KEGG pathways of upregulated DEGs and downregulated DEGs. Red indicated up-regulation, and green indicated down-regulated. Black circular nodes(count) represent the number of DEGs enriched in each term.

To deep mine the underlying mechanisms of the role of GMFB in HCC, LinkedOmics was utilized to analyze mRNA sequencing information from HCC patients in the TCGA. The plot showed the distribution of significant positive correlation with GMFB (red dots) or significant negative correlation (blue dots) in HCC patients (**Figure 4A**). Heat maps of the top 50 most positively and negatively co-regulated with GMFB in HCC patients were shown in **Figures 4B, C**. There were 3389 up-regulated genes and 1397 down-regulated genes in high expression level of GMFB in HCC patients (FDR (Benjamini & Hochberg (BH)-corrected p-value) <0.0001, P<0.0001) (**Supplementary Table 4**). GO (BP, MF, and CC) and KEGG enrichment analyses of co-expression genes were performed. As shown in **Figure 4D**, we found that the down-regulated co-expression genes of GMFB were associated with the mitochondrial electron transport chain and translation initiation, and the up-regulated co-expression genes of GMFB were relevant to transcription, DNA-template (BP), nucleoplasm (CC), protein binding (MF) and pathway in cancer (KEGG). The detailed information for the co-expression genes was obtained in **Supplementary Tables 5**, **6**. Furthermore, results of Kaplan Meier survival analysis (males and females were shown separately) and reports associated with HCC of TOP100 DEGs in overlap, male-specific and female-specific groups were summarized in **Supplementary Table 7**.

### Functional Enrichment Analysis of GMFB in Patients With HCC Based on Gender

**Figure 5:**
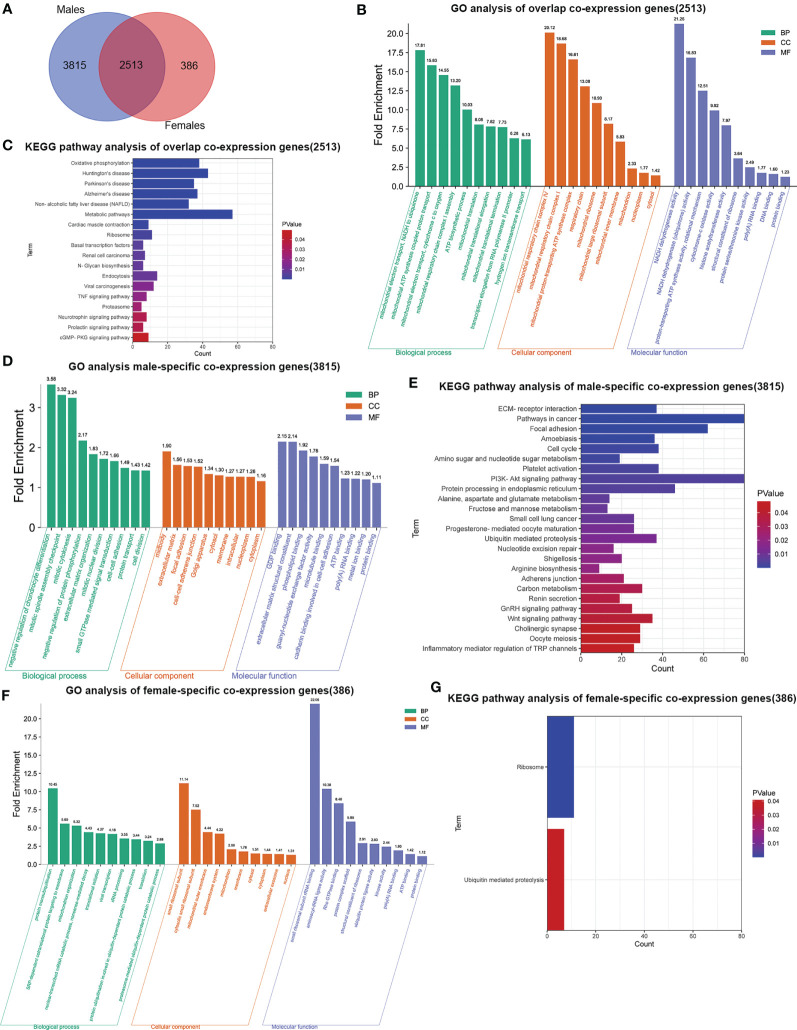
Sex differences in significant GO terms and KEGG pathways analysis. **(A)** Venn diagrams show the number of distinct and overlapping DEGs in male and female HCC patient group. **(B)** Enriched BP, CC and MF GO terms of 2513 overlap co-expression genes. Vertical axis represents GO terms. Fold enrichment was marked on the top of column. **(C)**. Significant KEGG pathways of 2513 overlap co-expression genes, P values are marked in different colors. **(D)** Enriched BP, CC and MF GO terms in 3815 male-specific co-expression genes. **(E)** Significant KEGG pathways of 3815 male-specific co-expression genes. **(F)** Enriched BP, CC and MF GO terms in 386 female-specific co-expression genes. **(G)** Significant KEGG pathways of 386 female-specific co-expression genes.

**Figure 6:**
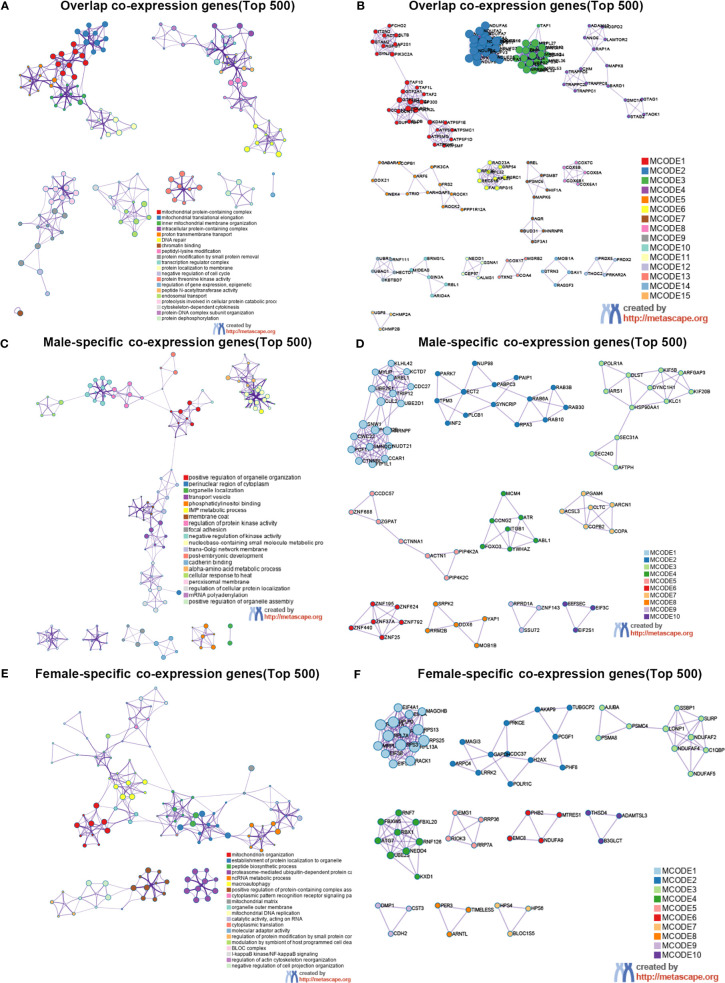
Sex differences in the enrichment analysis of GMFB in HCC (Metascape). Network of enriched terms of top 500 overlap co-expression genes **(A)**, male-specific co-expression genes **(C)** and female-specific co-expression genes **(E)**, and colored by cluster ID. PPI network of proteins of top 500 overlap co-expression genes **(B)**, male-specific co-expression genes **(D)** and female-specific co-expression genes **(F)**.

To elucidate the possible mechanism of underling GMFB expression level on the clinical outcome in HCC patients with different gender, male and female common, male-specific and female-specific GMFB co-expression genes were further analyzed. Venn diagram of GMFB co-expression genes (FDR (BH-corrected p-value) <0.01, P<0.01) in male and female HCC groups was illustrated that there were a larger number of regulated genes in males (6328 DEGs) than in females (2899 DEGs), and showing a substantial overlap (2513 DEGs) between males and females (**Figure 5A**). DAVID analysis software was utilized to identified functional enrichment terms, including GO (BP, MF, and CC) and KEGG of GMFB co-expression genes in overlap (**Figures 5B, C**), male-specific (**Figures 5D, E**) and female-specific (**Figures 5F, G**), respectively. The enrichment analysis disclosed the distinct role of GMFB co-expression genes in female and male HCC patients, despite increased expression of GMFB was observed in both male and female HCC patients. Results also showed a high expression level of GMFB related to mitochondrial dynamics. To further capture the relationships between the terms, PPI network and MCODE component analysis of top 500 DEGs of overlap (**Figures 6A, B**), male-specific (**Figures 6C, D**) and female-only (**Figures 6E, F**) were performed.

Regardless of gender, co-expression genes of GMFB were mainly involved in oxidative phosphorylation and mitochondria function (**Figures 6A, B**). In male patients, peroxisome, cell cycle and inosine monophosphate biosynthesis, PRPP + glutamine => IMP were enriched from male-specific co-expression genes of GMFB (**Figures 6C, D**) while in female patients, KEGG pathways such as ribosome and ubiquitin mediated proteolysis were enriched (**Figures 6E, F**). Detailed information was available in **Supplementary Table 8**. Likewise, regardless of gender, PPI networks showed that GO annotation mainly related to MCODE 1 were mitochondrial complex and transferase complex. In males, GO annotation is mainly related to MCODE 1 of mRNA splicing, *via* spliceosome, RNA splicing, *via* transesterification reactions with bulged adenosine as nucleophile and RNA splicing, *via* transesterification reactions, while in females, MCODE 1 was anchoring translation, peptide biosynthetic process and ribosomal subunit. **Supplementary Material** reports detailed data (**Supplementary Table 9**).

### The Increased mtDNA Copy Number and Loss of Mitochondrial Membrane Potential in Hep3B by Knocking Down GMFB

**Figure 7:**
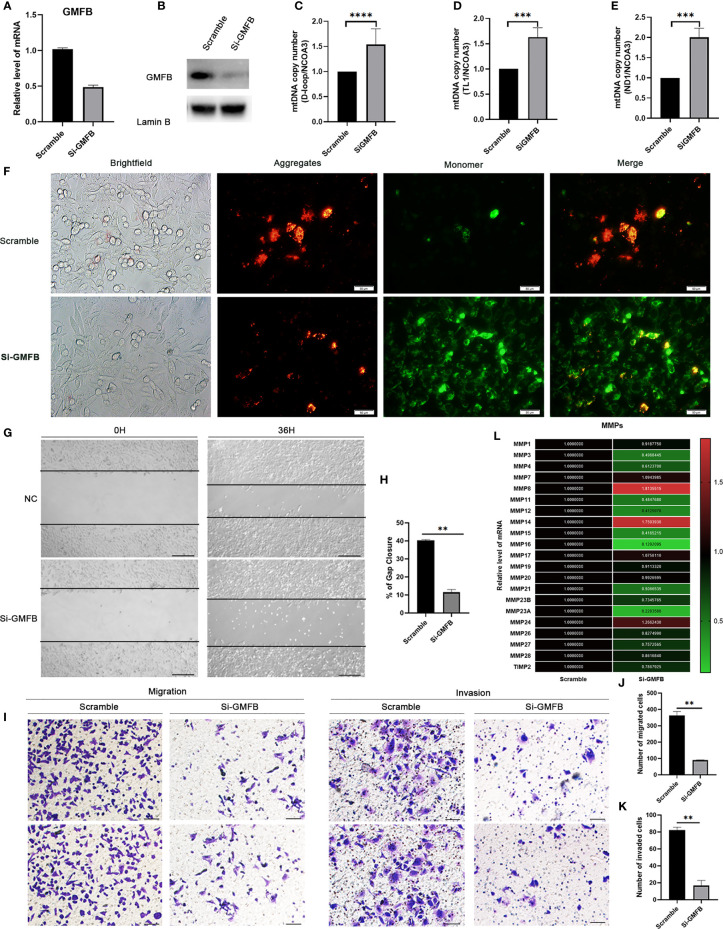
Knock down GMFB decreased mtDNA copy number and inhibits proliferation and invasion of Hep3B cell. **(A)** SiRNA efficiently knock down GMFB mRNA levels by qRT-PCR in Hep3B cells. **(B)** SiRNA efficiently knock down GMFB protein levels by western blot in Hep3B cells. **(C–E)** Relative mtDNA copy number (mtDNA/nDNA) was detected by qPCR and normalized by nuclear receptor coactivator 3(NCOA3) gene in knockdown GMFB. GMFB knockdown. **(F)** Fluorescence microscopic imaging of Hep3B cells stained with JC-1. Scale bar, 50 μm. **(G)** Image of the scratch assay. Scale bar, 100 μm. **(H)** Quantification of the scratch assay. Error bars represent standard error of means (SEM). **(I)** Transwell migration and invasion assays were used to examine the migration and invasion ability of HCC cells. Scale bar, 100 μm. Quantification of migration **(J)** and invasion assays **(K)**. Error bars represent standard error of means (SEM). **(L)** Heat map of relative qPCR results of MMPs. **P < 0.01, ***P < 0.001, ****P < 0.0001.

Based on the bioinformatic analysis above, mitochondria were identified to be a major target of GMFB and GMFB co-expression genes. Hence, the expression levels of mitochondrial expansion program and homeostasis-associated genes were analyzed. Results revealed that 52 genes required for mitochondrial ribosome function were down-regulated (**Table 1**). Mitochondrial dynamics-associated genes (MTP18, FIS1), mitochondrial protein import-associated genes (TOMM40, TOMM7), mitophagy-associated genes (PINK1) and glucose metabolism-associated genes (FBP1, TPI1 and PCK1) were significantly down-regulated in HCC patients with high GMFB expression (**Table 1**). Finally, we designed siRNA oligos against GMFB to verify the finding from the bioinformatic analysis. After the determination of the knockdown efficiency of siRNA against GMFB (**Figures 7A, B**), mtDNA copy number was measured by qPCR. We found that the expression level of GMFB negatively co-expression to mtDNA copy number in Hep3B cells (**Figures 7C**–**E**). Furthermore, we also detected mitochondrial membrane potential by JC-1 assay. As shown in **Figure 7F**, the transition of red to green fluorescent signal was observed in GMFB knockdown HCC cells, which indicated the loss of mitochondrial membrane potential is associated with low expression levels of GMFB in Hep3B cells.

**Table 1:** Relative expression of mitochondrial and glycolysis-associated genes in patients with high GMFB expression.

Description	Query	Statistic	P-value	FDR (BH)
Mitochondrial protein synthesis	MRPL1	-0.141321338	0.007160143	0.016746243
	MRPL10	-0.105032788	0.046127911	0.083444214
	MRPL11	-0.311787832	1.40402E-09	1.36064E-08
	MRPL12	-0.411325456	3.58815E-16	1.52363E-14
	MRPL14	-0.382101015	5.3916E-14	1.32397E-12
	MRPL15	-0.119926057	0.022671632	0.045620446
	MRPL16	-0.269955944	1.90488E-07	1.2005E-06
	MRPL17	-0.298656993	7.14778E-09	6.03425E-08
	MRPL19	0.157548668	0.002683783	0.006989346
	MRPL2	-0.376971399	1.23596E-13	2.79072E-12
	MRPL20	-0.377361436	1.161E-13	2.63641E-12
	MRPL21	-0.296536472	9.22667E-09	7.60237E-08
	MRPL22	-0.340013844	3.20959E-11	4.42054E-10
	MRPL23	-0.361800315	1.32141E-12	2.39235E-11
	MRPL24	-0.32727754	1.85288E-10	2.16296E-09
	MRPL32	-0.264832174	3.2938E-07	1.99866E-06
	MRPL34	-0.364195064	9.16604E-13	1.70919E-11
	MRPL36	-0.319586768	5.13832E-10	5.49274E-09
	MRPL37	-0.256312337	7.98478E-07	4.44927E-06
	MRPL38	-0.452159392	1.36218E-19	1.48969E-17
	MRPL4	-0.318917541	5.60762E-10	5.93704E-09
	MRPL41	-0.504373553	1.08008E-24	5.00226E-22
	MRPL46	-0.229639252	1.04789E-05	4.68855E-05
	MRPL47	-0.261962532	4.45388E-07	2.62279E-06
	MRPL49	0.145262194	0.0056907	0.013647675
	MRPL53	-0.437150183	2.79E-18	2.09671E-16
	MRPL54	-0.475611761	8.9333E-22	1.83209E-19
	MRPL55	-0.407366684	7.28546E-16	2.89601E-14
	MRPS12	-0.419680399	7.8018E-17	4.04617E-15
	MRPS14	-0.1433601	0.006362258	0.015065919
	MRPS15	-0.404028697	1.31418E-15	4.81987E-14
	MRPS16	-0.350958594	6.66495E-12	1.0501E-10
	MRPS17	-0.268732399	2.17326E-07	1.35505E-06
	MRPS18A	-0.273086412	1.35554E-07	8.82209E-07
	MRPS18B	-0.115880152	0.027700048	0.054056487
	MRPS18C	-0.241995373	3.29881E-06	1.6322E-05
	MRPS2	-0.404723616	1.16293E-15	4.33704E-14
	MRPS22	-0.181553225	0.000527481	0.00163143
	MRPS24	-0.534918562	4.13643E-28	4.11885E-25
	MRPS25	-0.45798162	4.0535E-20	4.98305E-18
	MRPS26	-0.438152315	2.29116E-18	1.76172E-16
	MRPS28	-0.110719774	0.035478952	0.066801865
	MRPS28	-0.110719774	0.035478952	0.066801865
	MRPS30	-0.136957465	0.009175224	0.020847072
	MRPS31	-0.103676942	0.049029226	0.087894232
	MRPS33	-0.363111917	1.08192E-12	1.98585E-11
	MRPS34	-0.387347676	2.27307E-14	6.07627E-13
	MRPS5	-0.358159625	2.29094E-12	3.95922E-11
	MRPS6	-0.176539551	0.000754091	0.002254576
	MRPS7	-0.291364752	1.70489E-08	1.33149E-07
	MRPS9	-0.249646084	1.56238E-06	8.2184E-06
Mitochondrial dynamics	MTP18(MTFP1)	-0.22064897	2.34E-05	9.74E-05
	FIS1	-0.399647594	2.82E-15	9.49E-14
Mitochondrial protein import	TOMM40	-0.277369089	8.45E-08	5.78E-07
	TOMM7	-0.423363936	3.93E-17	2.13E-15
Mitophagy	PINK1	-0.159223311	0.002412305	0.006368958
Glucose metabolism	FBP1	-0.218488252	2.82E-05	0.000115938
	TPI1	-0.245741921	2.29E-06	1.17E-05
	PCK1	-0.191431015	0.00025373	0.00084683

MRPLs, Mitochondrial Ribosomal Protein Ls; MRPSs, Mitochondrial Ribosomal Protein Ss; MTP18(MTFP1), Mitochondrial Fission Process 1; FIS1,Fission, Mitochondrial 1; TOMMs, Translocase Of Outer Mitochondrial Membranes; PINK1, PTEN Induced Kinase 1; FBP1, Fructose-Bisphosphatase 1; TPI1, Triosephosphate Isomerase 1; PCK1,Phosphoenolpyruvate Carboxykinase 1.

### The Inhibition of Hep3B Cell Proliferation, Migration and Invasion by GMFB Knockdown

To further verify the relationship between GMFB and the malignant behavior of the HCC, we also determined cell proliferation, invasive and migratory abilities by wound-healing and Transwell assays. Post-transfection 48 hours of siGMFB oligos in Hep3B cells, we found that decreased expression of GMFB inhibited wound closure significantly (**Figures 7G, H**). Transwell migration assay showed less motile with crystal violet staining in GMFB knockdown Hep3B cells (**Figures 7I, J**). Transwell-matrigel invasion assays showed that GMFB knockdown inhibited Hep3B invasion (**Figures 7I, K**). As shown in **Table 2**, MMPs expression patterns showed a gender disparity. There were more up-regulated MMPs appeared in the list of co-expression genes of GMFB in male HCC patients than in females. In females, the expression of MMP10, MMP12, MMP16, MMP23A were up-regulated while MMP19 and MMP24 were downregulated. In males, MMP1, MMP2, MMP3, MMP7, MMP8, MMP9, MMP10, MMP11, MMP12, MMP14, MMP16, MMP27 were up-regulated while MMP19 and MMP24 were downregulated. A validation experiment was conducted to examine MMPs expression after knockdown GMFB in Hep3B. qRT-PCR result showed that the expression levels of MMPs (except for MMP7, MMP8, MMP14, MMP17and MMP24) were decreased significantly after GMFB knockdown in Hep3B cells. Compared with MMPs expression patterns in male HCC patients, only MMP7, MMP8 and MMP14 expression levels were not consistent with bioinformatic analysis. Compared with MMPs expression patterns in female HCC patients, MMP12, MMP16, MMP23A and MMP24 expression levels were consistent with bioinformatic analysis. Taken together, our results supported that knockdown of GMFB suppressed the proliferation, invasion and migration of Hep3B cells, probably due to modulating MMPs expression.

**Table 2:** Relationship between MMPs and GMFB in male and female HCC.

Gene	Female		Male	
	Statistic	P-value	Statistic	P-value
MMP1	NS	NS	0.18	0
MMP2	NS	NS	0.22	0
MMP3	NS	NS	0.18	0
MMP7	NS	NS	0.29	0
MMP8	NS	NS	0.17	0.01
MMP9	NS	NS	0.14	0.02
MMP10	0.19	0.03	0.19	0
MMP11	NS	NS	0.13	0.04
MMP12	0.34	0	0.34	0
MMP14	NS	NS	0.28	0
MMP16	0.34	0	0.29	0
MMP19	-0.18	0.04	-0.23	0
MMP23A	0.19	0.04	NS	NS
MMP24	-0.26	0	-0.32	0
MMP27	NS	NS	0.12	0.05

MMP, Matrix metalloproteinases.

## Discussion

In the present study, we performed a comprehensive bioinformatic analysis on the association between GMFB and HCC. We found that GMFB was highly expressed in several types of cancer including HCC (**Figures 1B, C**, **2A**), and confirmed the expression level of GMFB was closely associated with the TNM stage, clinical stage, and poor survival rates in HCC (**Figures 1D, E**). GMFB was considered to be a novel potential biomarker and therapeutic target HCC (**Figure 3**). Moreover, knockdown of GMFB with siRNA effectively inhibited the proliferation, migration and invasion in Hep3B cells and down-regulated the expression level of some of the MMPs. Targeting GMFB may represent a promising therapeutic strategy for HCC. However, the potential mechanisms underlying GMFB contributing to HCC remain unclear.

To clarify the potential mechanism, we found a large number of DEGs were co-expressed with the high level of GMFB, and most of them were highly associated with mitochondrial functions (**Figure 4D**). Thus, we compared transcriptional profiles of DEGs related to mitochondrial protein synthesis (MRPLs and, MRPSs), Mitochondrial dynamics (MTP18 and FIS1), Mitochondrial protein import (TOMM40 and TOMM7), mitophagy (PINK1) and glucose metabolism (FBP1, TPI1 and PCK1) (44). The result indicated that a high level of GMFB down-regulated 53 genes required for mitochondrial ribosome function (**Table 1**), suggesting that the high level of GMFB may suppress mtDNA replication, mitophagy, and energy metabolism in HCC.

Increased expression of GMFB rather than AFP was an independent risk factor for HCC (**Figure 3E**). Moreover, knockdown of GMFB with siRNA effectively inhibited the proliferation, migration and invasion in Hep3B cells and down-regulated the expression level of some of the MMPs.

The mtDNA copy number gained more and more attention in cancer research. Previous studies showed decreased copy numbers in cancer samples in HCC, bladder cancer, breast cancer, kidney clear cell carcinoma and myeloproliferative neoplasm, and increased copy number detected in chronic lymphocytic leukemia, lung squamous cell carcinoma and pancreatic adenocarcinoma (45–50). Furthermore, the decrease in mtDNA copy number was more significant in female HCC patients than in males (51). In our study, we found that the decreased expression of GMFB leads to up-regulation of mtDNA copy number, which means the higher level of GMFB negatively regulates the amount of mtDNA copy number (**Figures 3C**–**E**). Moreover, our result also suggested that low expression levels of GMFB induced loss of mitochondrial membrane potential in Hep3b cells (**Figure 7F**). Previous works showed that mitochondria is a central regulator of the decision between cellular survival and demise (52). Mitochondrial membrane potential is an indicator of mitochondrial function, and loss of mitochondrial membrane potential resulting in the intrinsic apoptotic pathway (53). To date, there is no study has explored the role of GMFB in mitochondrial dysfunction. which might partially contribute to the malignancy of HCC. Therefore, these results suggested that GMFB may regulate mtDNA function and consequently be involved in HCC tumorigenesis and progression.

It is well known that cancer cells are commonly exposed to nutritional deficiency and hypoxia, and these factors adversely impact cancer cell metastasis (54). Cancer cells use aerobic glycolysis to support proliferation instead of mitochondrial oxidative phosphorylation (55). Therefore, inhibition glycolysis in cancer cells represents a kind of therapeutic strategy (56). In our study, GMFB down-regulated the expression level of the negative regulator of glycolysis, fructose-1,6-bisphosphatase (FBP1), Triosephosphate isomerase (TPI1) and phosphoenolpyruvate carboxykinase 1 (PCK1) (**Table 1**). This suggests that high expression levels of GMFB co-expression with the promotion of glycolysis and suppression of gluconeogenesis. Furthermore, previous works showed that suppressed FBP1, a negative regulator of aerobic glycolysis, led to promoting HCC growth and metastasis (57). Knockout PCK1, a step limiting enzyme of gluconeogenesis, markedly enhanced the global O-GlcNAcylation levels, which is an emerging hallmark of HCC (58). TPI1 functioned as a tumor suppressor in HCC (59). In conjunction with down-regulated KEGG pathways from co-expression genes of GMFB, we favored that targeting GMFB/mtDNA/glycolysis may represent a novel therapeutic strategy.

According to epidemiological and clinical characteristics of HCC, there is a remarkable gender disparity of HCC incidence, primarily males. Liver cancer ranks fifth in terms of global cases and second in terms of deaths for males. Women appeared to have better survival rates than men in HCC (60). In 2018, the distribution of cases in both sexes, males, and females were 6th, 5th and 9th, and for deaths is 4th, 2nd and 6th respectively (1). When we did KM Plotter survival analysis, we found that high GMFB expression was significantly associated with poor OS of HCC patients (P=0.0042) (**Figure 3A**). Unexpectedly, there was a significant OS difference between male patients with low and high expression of GMFB (P=0.00014) (**Figure 3B**). However, it did not occur in females with HCC (P=0.36) (**Figure 3C**).

It is widely recognized that the liver is one of the main responsible organs for estrogens (61, 62). Previous studies indicate that estrogen and estrogen receptors played a role in the development and progression of HCC (62–64). But, the role of estrogen and estrogen receptors of HCC is disputed (65). It seems that increased estrogen synthesis and variants estrogen receptor in liver leads to an increased risk of HCC (66, 67). On the contrary, estrogen and estrogen receptors can decrease the malignancy of HCC by arresting cell cycle progression and promoting apoptosis (68). In addition, distinct gender differences were observed in our study. GO and KEGG pathways enrichment analyses of DEGs (**Figures 5**, **6**). Further analysis showed that more DEGs co-expressed with GMFB were regulated in the males than in the females (**Figure 5A**). Results of survival analysis and current research of top 100 DEGs were shown in **Supplementary Table 7**. These distinct results above indicated that sex hormones might play a key role in regulating the gene expression profiling of co-expression genes of GMFB. Among top 100DEGs, some of them with statistical significance of survival analysis, such as Dodecenoyl-CoA isomerase (DCI, p value=4.3e-5 in males), G2/M phase-specific E3 ubiquitin-protein ligase (G2E3, p value=9.5e-6 in males), LSM domain-containing protein 1 (LSMD1, p value=0.0001 in males), Neural precursor cell expressed developmentally down-regulated protein 1 (NEDD1, p value=0.0001 in males) and Ras-related GTP-binding protein C (RRAGC, p value=0.0001 in females) have not been reported in HCC yet. These genes merit further investigation.

It is well known that the MMPs play a vital role in cancer invasion and metastasis (69). We also found that expression patterns of co-expression MMPs genes of GMFB showed a gender disparity (**Table 2**), which may explain the significant correlation between OS and GMFB in male HCC, not in females. Knockdown of GMFB effectively inhibited the expression levels of MMPs. Our results supported that the expression level of GMFB in male HCC modulated a subset of MMPs expression, finally contributing to male OS.

Certain limitations exist in our study. The first limitation is that we lack validation of our novel findings with human HCC samples. Since we could not obtain the levels of sex hormones, the expression level of sex hormone receptors in HCC patients used in this study, the relationship between levels of sex hormones, sex hormones reporters and GMFB expression merits further investigation. The second limitation is that mechanistic study is preliminary and should be deeply explored.

In summary, this work will shed deep light onto GMFB function in HCC. Targeting GMFB may represent a promising therapeutic strategy for HCC patients. GMFB may be an invaluable diagnostic and prognostic biomarker for HCC from the perspective of precision medicine.

## Data Availability Statement

Publicly available datasets analyzed in this study can be found here: http://www.oncomine.org; http://ualcan.path.uab.edu; https://www.Proteinatlas.org; http://www.cbioportal.org/; http://www.linkedomics.org; http://kmplot.com/analysis/. The original contributions presented in the study are included in the article/**Supplementary Material**. Further inquiries can be directed to the corresponding authors.

## Author Contributions

Among the authors in the list, WS designed the study, performed experiments and wrote the manuscript. CH performed part of data analysis and interpreted the experimental data. TW performed part of data analysis. JW, JPZ, FG, QO, HT, CJ, JX, and JFZ discussed the results. LL and G-TX encouraged us to investigate and supervised the findings of this work. All authors contributed to the article and approved the submitted version.

## Funding

This work was supported by grants obtained from the Ministry of Science and Technology of China (2017YFA0104100), and the National Natural Science Foundation (81670867, 81372071, 81770942).

## Conflict of Interest

The authors declare that the research was conducted in the absence of any commercial or financial relationships that could be construed as a potential conflict of interest.

## Publisher’s Note

All claims expressed in this article are solely those of the authors and do not necessarily represent those of their affiliated organizations, or those of the publisher, the editors and the reviewers. Any product that may be evaluated in this article, or claim that may be made by its manufacturer, is not guaranteed or endorsed by the publisher.
